# Hospital admissions for vitamin D related conditions and subsequent immune-mediated disease: record-linkage studies

**DOI:** 10.1186/1741-7015-11-171

**Published:** 2013-07-25

**Authors:** Sreeram V Ramagopalan, Raph Goldacre, Giulio Disanto, Gavin Giovannoni, Michael J Goldacre

**Affiliations:** 1 Department of Physiology, Anatomy and Genetics and Medical Research Council Functional Genomics Unit, University of Oxford, Oxford, UK; 2 Blizard Institute, Barts and the London School of Medicine and Dentistry, Queen Mary University of London, London, UK; 3 Unit of Health-Care Epidemiology, Nuffield Department of Population Health, University of Oxford, Oxford, UK

**Keywords:** Vitamin D, Immune disease, Hospital episode statistics

## Abstract

**Background:**

Previous studies have suggested that there may be an association between vitamin D deficiency and the risk of developing immune-mediated diseases.

**Methods:**

We analyzed a database of linked statistical records of hospital admissions and death registrations for the whole of England (from 1999 to 2011). Rate ratios for immune-mediated disease were determined, comparing vitamin D deficient cohorts (individuals admitted for vitamin D deficiency or markers of vitamin D deficiency) with comparison cohorts.

**Results:**

After hospital admission for either vitamin D deficiency, osteomalacia or rickets, there were significantly elevated rates of Addison’s disease, ankylosing spondylitis, autoimmune hemolytic anemia, chronic active hepatitis, celiac disease, Crohn’s disease, diabetes mellitus, pemphigoid, pernicious anemia, primary biliary cirrhosis, rheumatoid arthritis, Sjogren’s syndrome, systemic lupus erythematosus, thyrotoxicosis, and significantly reduced risks for asthma and myxoedema.

**Conclusions:**

This study shows that patients with vitamin D deficiency may have an increased risk of developing some immune-mediated diseases, although we cannot rule out reverse causality or confounding. Further study of these associations is warranted and these data may aid further public health studies.

## Background

Immune-mediated diseases cumulatively represent one of the most common chronic disease groups in medicine today, affecting approximately 10% of first world populations [1]. There is substantial evidence in support of these disorders being determined by both genetic and environmental factors. One candidate environmental risk factor implicated in immune-mediated disease susceptibility is vitamin D deficiency [2-4].

Historically, vitamin D was thought to play a restricted role in calcium homeostasis; however, a wealth of studies now suggests that it exerts more widespread effects [5,6]. Functional laboratory studies have shown that vitamin D can modulate the immune response [5,6] and a number of epidemiological findings have implicated the involvement of vitamin D deficiency in the risk of developing immune-mediated diseases [2-4]. These include the ecological findings of the prevalence of diseases, such as multiple sclerosis (MS), type 1 diabetes, inflammatory bowel disease, rheumatoid arthritis and Sjogren’s syndrome, positively correlating with latitude and reduced ultraviolet radiation exposure (the primary determinant of vitamin D levels) [1]. Further support comes from studies showing low serum vitamin D levels in patients suffering from a wide range of immune disorders, including MS [7], type 1 diabetes [8], systemic lupus erythematosus (SLE) [9] and rheumatoid arthritis [10]. These findings may, however, be biased by reverse causation [11].

To investigate further any association between vitamin D and immune-mediated disease, we undertook record linkage studies to determine the risk of immune-mediated disease in individuals admitted for vitamin D deficiency or a marker of vitamin D deficiency (rickets or osteomalacia) using an English national linked Hospital Episode Statistics (HES) dataset.

## Methods

### Population and data

We used a linked English national dataset of hospital admissions (Hospital Episode Statistics (HES)) and mortality. HES data are records of hospital care that are compiled for every episode of day case care or hospital admission in all English National Health Service (NHS) hospitals, and were supplied by the English national Information Centre for Health and Social Care. The mortality data were derived from death certificates and were supplied by the Office for National Statistics. The linked dataset used in this study, in which successive records for each individual were linked together, was constructed by the Oxford record linkage group.

The International Classification of Disease (ICD) codes used for the vitamin D related conditions were osteomalacia (M83.1, M83.8, M83.9), rickets (E55.0, E64.3) and vitamin D deficiency (E55.9). The ICD codes used for the immune-mediated diseases were Addison’s disease (E27.1), ankylosing spondylitis (M45), asthma (J45), autoimmune hemolytic anemia (D59.1), chronic active hepatitis (K73.2), Crohn’s disease (K50), celiac disease (K90.0), dermatomyositis (M33.0 to M33.1), diabetes mellitus (E10 to E14), polymyositis (M33.2), Goodpasture’s syndrome (M31.0), Hashimoto’s thyroiditis (E06.3), idiopathic thrombocytopenia purpura (D69.3), multiple sclerosis (G35), myasthenia gravis (G70.0), myxoedema (E03.8 to E03.9), pemphigus (L10), pemphigoid (L12), pernicious anemia (D51.0), polyarteritis nodosa (M30.0), primary biliary cirrhosis (K74.3), psoriasis (L40), rheumatoid arthritis (M05 to M06), scleroderma (M34), Sjogren’s syndrome (M35.0), SLE (M32.1 to M32.9), thyrotoxicosis (E05), and ulcerative colitis (K51). In the analysis of diabetes mellitus, we used hospital admission for diabetes mellitus when the patient was aged under 30 as a proxy for type 1 diabetes, as the type of diabetes is not well recorded in routine hospital statistics. We also confined the analysis of asthma admissions to people aged between the ages of 5 and 54 to reduce potential issues with misclassification of asthma-like respiratory conditions in younger and older people.

The methods of analysis were the same for all vitamin D-related admissions and immune-mediated diseases; we describe the methods for rickets and Crohn’s disease as the example. A cohort of people with rickets was constructed for those with a diagnosis of rickets as a reason for hospital care, by identifying the first episode of day case care, or admission, for rickets during the study period. A reference cohort was constructed by identifying the first admission for each individual with various other, mainly minor medical and surgical, conditions (listed in the Table 1 legend), as in previous studies of disease associations [12]. Standard epidemiological practice was followed by selecting a diverse range of conditions rather than relying on a limited range (in case the latter are themselves atypical in their risk of immune-mediated disease) [12]. As a check, we studied the risk of immune-mediated disease in the control conditions within the reference cohort to ensure that the reference cohort did not include control conditions that have atypically high or low immune mediated-disease rates. For some immune-mediated diseases (for example, rheumatoid arthritis), we removed some control conditions (for example, hip/knee replacement) from the reference cohort where we considered that this may otherwise have skewed the findings. For the fairly small number of associations affected by this, we show the ‘adjusted’ associations (that is, with some control conditions removed) for these diseases in the main paper; for completeness and comparison, the unadjusted associations are also provided (see below). Anyone with both an ‘exposure’ disease, for example, Crohn’s disease, and a reference cohort condition was included in the exposure cohort and excluded from the reference cohort.

**Table 1:** **Age-distribution of people in exposure cohorts, percentage who were female, and numbers in reference cohort**^**1**^

**Exposure condition**	**Age at admission**	**N in the exposure cohort (% of total)**	**% female**	**Number in the reference cohort**^**2**^
Rickets, osteomalacia, or vitamin D deficiency, all combined:	<15	745 (7.5)	53.0	889,364
	15 to 44	532 (24)	72.4	2,906,048
	45 to 64	1,191 (26.7)	70.4	2,206,093
	65 to 74	1,356 (15.1)	68.9	1,198,951
	75+	1,697 (26.7)	73.1	1,403,767
	All ages	19,338 (100)	70.1	8,604,223
Rickets:	<15	494 (54.8)	48.9	889,495
	15 to 44	73 (16.8)	70.4	2,907,044
	45 to 64	32 (8.7)	71.0	2,207,541
	65 to 74	29 (7)	68.6	1,200,051
	75+	42 (12.7)	78.2	1,405,341
	All ages	1,228 (100)	59.5	8,609,472
Osteomalacia:	<15	1 (0.1)	0.0	889,562
	15 to 44	39 (16.9)	68.8	2,906,823
	45 to 64	275 (30.3)	62.7	2,207,108
	65 to 74	503 (20.5)	68.3	1,199,703
	75+	585 (32.2)	76.7	1,404,882
	All ages	5,191 (100)	69.4	8,608,078
Vitamin D deficiency:	<15	263 (6)	56.8	889,428
	15 to 44	436 (27.5)	73.5	2,906,311
	45 to 64	911 (26.9)	74.0	2,206,566
	65 to 74	847 (13.8)	69.5	1,199,332
	75+	1,107 (25.8)	71.5	1,404,315
	All ages	13,260 (100)	71.5	8,605,952

^1^ The reference cohort consisted of people admitted with the following conditions coded under the Office of Population, Censuses and Surveys (OPCS) code edition 4 for operations and ICD10 code for diagnosis (with equivalent codes used for other coding editions): appendectomy (OPCS4 H01 to H03), adenoidectomy (E20), tonsillectomy (F34 + F36), dilation and curettage (Q10.3 + Q11.4), total hip replacement (W37 to W39), total knee replacement (W40 to W42), squint (ICD10 H49 to H51), cataract (H25), otitis externa/media (H60 to H67), varicose veins (I83), hemorrhoids (I84), deflected septum, nasal polyp (J33 + J34.2), impacted tooth and other disorders of teeth (K00 to K03), inguinal hernia (K40), in-growing nail, toenail and other diseases of the nails (L60), sebaceous cyst (L72.1), bunion (M20.1), internal derangement of the knee (M23), dislocations, sprains and strains (S03, S13, S23, S33, S43, S53, S63, S73, S83, S93), selected limb fractures (S42, S52, S62, S82, S92), superficial injury and contusion (S00, S10, S20, S30, S40, S50, S60, S70, S80, S90), contraceptive management (Z30).

^2^ The numbers in the reference cohort vary slightly depending on what the exposure condition is. This is because individuals, who otherwise fulfilled the criteria for inclusion in the reference cohort, were removed from the reference cohort if they fulfilled the criteria for inclusion in the exposure cohort (see Methods).

People were included in the rickets or reference cohort if they did not have an admission for an immune-mediated disease either before or at the same time as the admission for rickets or the reference condition. The database was then investigated for any subsequent NHS hospital care for, or death from, Crohn’s disease in these cohorts. We considered that rates of Crohn’s disease in the reference cohort would approximate those in the general population while allowing for migration as data on migration of individuals were not available.

The analysis was performed using a suite of programs developed ‘in house’ using SAS 9 software (SAS Institute, Cary, NC, USA).

### Ethical approval

The construction and analysis of the datasets were undertaken with the approval of the Central and South Bristol Research Ethics Committee (REC, reference 04/Q2006/175).

### Statistical methods

Rates of Crohn’s disease were calculated based on person-years. Date of entry into each cohort was the date of the first admission for rickets, or reference condition, and date of exit was the date of the first record of Crohn’s disease, death or the end of data collection (28 February 2011), whichever was the earliest. We first calculated rates for Crohn’s disease, stratified and then standardized by age (in five-year age groups), sex, calendar year of first recorded admission, region of residence, and quintile of patients’ Index of Deprivation score (a standard English measure of socio-economic status). The indirect method of standardization was used, with the combined rickets and reference cohorts as the standard population. We applied the stratum-specific rates in the standard population to the number of people in each stratum in the rickets cohort and then, separately, to those in the same stratum in the reference cohort, to obtain the expected number of people with Crohn’s disease in each stratum of the rickets and reference cohort. Observed and expected numbers were then summed across all strata to give totals for all strata combined. Rate ratios were calculated by taking the standardized rate of occurrence of Crohn’s disease in the rickets cohort relative to the reference cohort using the formula (O^rickets^/E^rickets^)/(O^ref^/E^ref^), where O and E are the observed and expected numbers of Crohn’s disease cases in the rickets and reference cohorts, respectively. In each table, we only show diseases in which either the observed or expected number, or both were five or more. The confidence interval for the rate ratio of Crohn’s and χ^2^ statistics for its significance were calculated as described elsewhere [13].

## Results

The number of people in the cohort with vitamin D deficiency was 13,260 (71.5% female), osteomalacia 5,191 (69.4% female), and rickets 1,228 (59.5% female). Age distributions are shown in Table 1. There were more than 8.6 million people in the reference cohort.

There were significantly elevated risks of Addison’s disease, ankylosing spondylitis, autoimmune hemolytic anemia, chronic active hepatitis, celiac disease, Crohn’s disease, diabetes mellitus, pemphigoid, pernicious anemia, primary biliary cirrhosis, rheumatoid arthritis, Sjogren’s syndrome, systemic lupus erythematosus, thyrotoxicosis; and significantly reduced risks for asthma and myxoedema after hospital admission for either vitamin D deficiency, osteomalacia or rickets (Table 2).

**Table 2:** Rate ratios for immune-mediated diseases following admission for rickets, osteomalacia or vitamin D deficiency, combined

**Disease**	**O**	**E**	**RR (95% CI)**	***P*-value**
Addison’s disease	21	3	7.2 (4.4 to 11.0)	<0.001
Ankylosing spondylitis^1^	16	8.2	2.0 (1.1 to 3.2)	0.01
Asthma (aged 5 to 54)^2^	150	375.5	0.4 (0.3 to 0.5)	<0.001
Autoimmune hemolytic anemia	6	2.2	2.7 (1.0 to 5.9)	0.03
Chronic active hepatitis	6	1	6.2 (2.3 to 13.5)	<0.001
Celiac disease^3^	78	14.3	5.5 (4.3 to 6.9)	<0.001
Crohn’s disease^3^	47	17.9	2.6 (1.9 to 3.5)	<0.001
Diabetes mellitus (aged 0 to 29)^4^	7	1.8	3.9 (1.6 to 8.1)	<0.001
Hashimoto’s	6	2.8	2.1 (0.8 to 4.6)	0.11
Idiopathic thrombocyt. purpura	11	8.3	1.3 (0.7 to 2.4)	0.44
Multiple sclerosis^5^	15	13.1	1.1 (0.6 to 1.9)	0.7
Myxoedema	335	520.4	0.6 (0.6 to 0.7)	<0.001
Pemphigoid	13	4.9	2.7 (1.4 to 4.5)	0.001
Pernicious anemia	60	35.7	1.7 (1.3 to 2.2)	<0.001
Polymyositis	5	1	5.1 (1.6 to 11.9)	<0.001
Primary biliary cirrhosis	12	4.4	2.8 (1.4 to 4.8)	0.001
Psoriasis	29	44.2	0.7 (0.4 to 0.9)	0.03
Rheumatoid arthritis^1^	158	126.2	1.3 (1.1 to 1.5)	0.005
Scleroderma	6	3.4	1.8 (0.7 to 3.9)	0.25
Sjogren’s syndrome	20	9.4	2.1 (1.3 to 3.3)	0.001
Systemic lupus erythematosus	33	8.1	4.1 (2.8 to 5.8)	<0.001
Thyrotoxicosis	100	57.7	1.7 (1.4 to 2.1)	<0.001
Ulcerative colitis^3^	32	25.2	1.3 (0.9 to 1.8)	0.21

*O* Observed number of cases, *E* Expected number, *RR* Rate ratio, *95% CI* 95% Confidence interval.

^1^Hip replacement and knee replacement were excluded from the reference cohort.

^2^Nasal polyp and deflected septum were excluded from the reference cohort.

^3^Hemorrhoids, appendectomy and cholelithiasis were excluded from the reference cohort.

^4^Cataracts were excluded from the reference cohort.

^5^Limb fractures, dislocations, superficial injury, squint and head injury were excluded from the reference cohort.

Diseases studied, but with fewer than five observed cases: dermatomyositis, Goodpasture’s syndrome, myasthenia gravis, pemphigus, polyarteritis nodosa.

There were significantly elevated risks of celiac disease, pernicious anemia and thyrotoxicosis after hospital admission for rickets (Table 3). Celiac disease had a substantially increased risk.

**Table 3:** Rate ratios for immune-mediated diseases following admission for rickets

**Disease**	**O**	**E**	**RR (95% CI)**	***P*-value**
Asthma (aged 5 to 54)	21	32.7	0.6 (0.4 to 1.0)	0.05
Celiac disease	10	1	10.3 (4.9 to 18.9)	<0.001
Myxoedema	26	17.7	1.5 (1.0 to 2.2)	0.06
Pernicious anemia	6	1.6	3.7 (1.4 to 8.0)	0.002
Rheumatoid arthritis	9	5	1.8 (0.8 to 3.4)	0.12
Thyrotoxicosis	7	2.1	3.4 (1.4 to 6.9)	0.002

*O* Observed number of cases; *E* Expected number, *RR* Rate ratio, *95% CI* 95% confidence interval.

There were significantly elevated risks of Addison’s disease, celiac disease, diabetes mellitus, Sjogren’s syndrome and thyrotoxicosis; and significantly reduced risks for asthma and myxoedema after hospital admission for osteomalacia (Table 4). The rate ratios for Addison’s disease, and celiac disease were particularly high. There were significantly elevated rates of Addison’s disease, celiac disease, Crohn’s disease, diabetes mellitus, pemphigoid, pernicious anemia, primary biliary cirrhosis, rheumatoid arthritis, Sjogren’s syndrome, SLE and thyrotoxicosis; and significantly reduced rates for asthma and myxoedema after hospital admission for vitamin D deficiency (Table 5). Again, the rate ratios for Addison’s disease and celiac disease were particularly high, as was the rate ratio for SLE.

**Table 4:** Rate ratios for immune-mediated diseases following admission for osteomalacia

**Disease**	**O**	**E**	**RR (95% CI)**	***P*-value**
Addison’s disease	9	1.1	8.1 (3.7 to 15.4)	<0.001
Ankylosing spondylitis	5	2.9	1.7 (0.57 to 4.1)	0.33
Asthma (aged 5 to 54)	50	71.9	0.7 (0.5 to 0.9)	0.01
Celiac disease	32	4.9	6.5 (4.4 to 9.2)	<0.001
Crohn’s disease	12	7.1	1.7 (0.9 to 3.0)	0.10
Diabetes mellitus (aged 0 to 29)	11	3.8	2.9 (1.5 to 5.2)	0.001
Idiopathic thrombocyt. purpura	5	2.9	1.8 (0.6 to 4.1)	0.33
Myxoedema	143	185.8	0.8 (0.7 to 0.9)	0.002
Pernicious anemia	23	15.1	1.5 (1.0 to 2.3)	0.06
Primary biliary cirrhosis	5	1.9	2.7 (0.9 to 6.3)	0.05
Psoriasis	11	14.6	0.8 (0.4 to 1.4)	0.42
Rheumatoid arthritis	54	49.3	1.1 (0.8 to 1.4)	0.55
Sjogren’s syndrome	8	3.7	2.2 (0.9 to 4.3)	0.04
Thyrotoxicosis	30	20.8	1.4 (1.0 to 2.1)	0.06
Ulcerative colitis	11	10.7	1.0 (0.5 to 1.9)	0.96

*O* Observed number of cases, *E* Expected number, *RR* Rate ratio, *95% CI* 95% confidence interval.

**Table 5:** Rate ratios for immune-mediated diseases following admission for vitamin D deficiency (coded as such)

**Autoimmune condition**	**O**	**E**	**RR (95% CI)**	***P*-value**
Addison’s disease	12	1.7	7.0 (3.6 to 12.3)	<0.001
Ankylosing spondylitis	10	5.3	1.9 (0.9 to 3.5)	0.06
Asthma (aged 5 to 54)	83	278.9	0.3 (0.2 to 0.4)	<0.001
Celiac disease	38	8.6	4.4 (3.1 to 6.1)	<0.001
Crohn’s disease	33	10	3.3 (2.3 to 4.6)	<0.001
Diabetes mellitus (aged 0 to 29)	20	6.2	3.2 (2.0 to 5.0)	<0.001
Idiopathic thrombocyt. purpura	5	5	1.0 (0.3 to 2.3)	0.83
Multiple sclerosis	12	8.2	1.5 (0.8 to 2.6)	0.25
Myxoedema	177	328.2	0.5 (0.5 to 0.6)	<0.001
Pemphigoid	10	2.7	3.7 (1.8 to 6.8)	<0.001
Pernicious anemia	34	19.6	1.7 (1.2 to 2.4)	0.002
Primary biliary cirrhosis	7	2.4	2.9 (1.2 to 6.0)	0.008
Psoriasis	18	28.9	0.6 (0.4 to 1.0)	0.05
Rheumatoid arthritis	101	74.5	1.4 (1.1 to 1.7)	0.003
Sjogren’s syndrome	12	5.7	2.1 (1.1 to 3.7)	0.01
Systemic lupus erythematosus	28	5.3	5.3 (3.5 to 7.7)	<0.001
Thyrotoxicosis	67	35.9	1.9 (1.5 to 2.4)	<0.001
Ulcerative colitis	21	13.6	1.5 (0.9 to 2.3)	0.06

*O* Observed number of cases, *E* Expected number, *RR* Rate ratio, *95% CI* 95% confidence interval.

Unadjusted associations when done, see Methods, are shown in Table 6.

**Table 6:** Unadjusted* RRs for immune-mediated diseases following admission for rickets, osteomalacia or vitamin D deficiency, combined

**Autoimmune condition**	**O**	**E**	**RR (95% CI)**	***P*-value**
Ankylosing spondylitis	16	9.3	1.73 (0.99 to 2.81)	0.04
Asthma (aged 5 to 54)	150	393	0.38 (0.32 to 0.45)	<0.001
Celiac disease	78	15.8	4.96 (3.91 to 6.19)	<0.001
Crohn’s disease	47	23.2	2.03 (1.49 to 2.7)	<0.001
Diabetes mellitus (aged 0 to 29)	20	6.3	3.2 (1.96 to 4.95)	<0.001
Multiple sclerosis	15	16.4	0.92 (0.51 to 1.51)	0.826
Rheumatoid arthritis	158	147.2	1.07 (0.91 to 1.25)	0.397
Ulcerative colitis	32	28.9	1.11 (0.76 to 1.56)	0.626

*O* Observed number of cases, *E* Expected number, *RR* Rate ratio, *95% CI* 95% confidence interval.

* See Methods section and legend for Table 2.

## Discussion

We present evidence associating vitamin D deficiency (or proxies of vitamin D deficiency) with risks of or protection against developing subsequent immune-mediated disease. Previous studies have linked inherited forms of rickets with MS [14] and type 1 diabetes [15], but no study has attempted an analysis similar to the one we present here. Addison’s disease and thyrotoxicosis were significantly associated in all analyses, and asthma was significantly low in all but one. The combined analysis found associations with a greater number of immune-mediated diseases - a lack of significance in the individual rickets, osteomalacia or vitamin D deficiency cohorts may reflect the lower power of these individual analyses.

The associations described warrant further attention. Mechanistically, it is possible that vitamin D deficiency predisposes to the development of immune-mediated disease by perturbing the immune response [2]. Another explanation is reverse causality - that the disease (either sub-clinically or clinically present but not recorded with a hospital admission prior to the vitamin D deficiency admission) reduces vitamin D levels as a result of an inflammatory state and/or causes the individual to spend less time outdoors [16]. The increasing awareness of vitamin D may also lead to an increase in requests for vitamin D testing, and subsequent diagnosis of vitamin D deficiency [17]. However, under either of these hypotheses the apparent protective effects of vitamin D deficiency on asthma and myxoedema are not easy to reconcile. There are reports, however, that state that higher levels of vitamin D in childhood may increase the risk of asthma [18,19]. For disorders such as celiac disease there may be an issue with regards to intake and/or malabsorption of vitamin D; with Addison’s disease, glucocorticoid deficiency may lead to suppression of the parathyroid hormone-vitamin D axis [20].

The study has limitations. It is not a prospective cohort with follow-up from the date of “first ever” diagnosis, but just from “first recorded” diagnosis in a hospital day case or inpatient record. Data are not recorded on patients who move out of the area covered by data collection or who are treated in hospitals outside the area. The dataset is limited to people who were admitted to hospital, or who received day case specialist care, and thus there exists the potential for selection bias. There is very limited information on potential confounding factors, such as detailed socioeconomic characteristics, ethnicity and smoking. The effect of making multiple comparisons needs to be considered. It is possible that some of the associations that are significant may result from making multiple comparisons and the play of chance. To aid assessment of this, we have provided *P*-values so that readers can judge where *P*-values are extreme: for example, with *P*-values as high as <0.001 for the associations between the vitamin D related conditions, combined, and celiac and Crohn’s disease, the associations are unlikely to be chance alone.

## Conclusions

Our results should be regarded as speculative. Further studies should look at individual immune-mediated diseases in greater depth to confirm or refute the findings and investigate mechanisms behind any association. Regardless of the direction of causality, the fact that individuals with immune-mediated disease are at risk of vitamin D deficiency is of note to ensure they are made replete to support adequate bone health [2].

## Abbreviations

HES: Hospital episode statistics; ICD: International classification of disease; NHS: National Health Service; OPCS: Office of population, censuses and surveys; SLE: Systemic lupus erythematosus

## Competing interests

The authors declared that they have no competing interest.

## Authors’ contributions

MJG is the guarantor and designer of the study. RG undertook the analysis. SVR and MJG contributed to the analysis and interpretation of the data. SVR wrote the first draft and all authors contributed to subsequent drafts and the final paper.

## Pre-publication history

The pre-publication history for this paper can be accessed here:

http://www.biomedcentral.com/1741-7015/11/171/prepub
